# The Grapevine Calmodulin-Like Protein Gene *CML21* Is Regulated by Alternative Splicing and Involved in Abiotic Stress Response

**DOI:** 10.3390/ijms21217939

**Published:** 2020-10-26

**Authors:** Olga A. Aleynova, Konstantin V. Kiselev, Zlata V. Ogneva, Alexandra S. Dubrovina

**Affiliations:** Laboratory of Biotechnology, Federal Scientific Center of the East Asia Terrestrial Biodiversity, Far Eastern Branch of the Russian Academy of Sciences, 690022 Vladivostok, Russia; aleynova@biosoil.ru (O.A.A.); kiselev@biosoil.ru (K.V.K.); zlata.v.ogneva@gmail.com (Z.V.O.)

**Keywords:** calcium sensor protein, cold stress, mRNA splice variants, transgenic plants and cell cultures, *Vitis amurensis*

## Abstract

Calmodulin-like proteins (CMLs) represent a large family of plant calcium sensor proteins involved in the regulation of plant responses to environmental cues and developmental processes. In the present work, we identified four alternatively spliced mRNA forms of the grapevine *CML21* gene that encoded proteins with distinct N-terminal regions. We studied the transcript abundance of *CML21v1*, *CML21v2, CML21v3*, and *CML21v4* in wild-growing grapevine *Vitis amurensis* Rupr. in response to desiccation, heat, cold, high salinity, and high mannitol stress using quantitative real-time RT-PCR. The levels of all four splice variants of *VaCML21* were highly induced in response to cold stress. In addition, *VaCML21v1* and *VaCML21v2* forms were highly modulated by all other abiotic stress treatments. Constitutive expression of *VaCML21v2* and *VaCML21v4* improved biomass accumulation of *V. amurensis* callus cell cultures under prolonged low temperature stress. Heterologous expression of the grapevine *CML21v2* and *VaCML21v4* splice variants in *Arabidopsis* improved survival rates of the transgenic plants after freezing. The *VaCML21v2* overexpression enhanced activation of the cold stress-responsive marker genes *AtDREB1A* and *AtDREB2A*, while *VaCML21v4* overexpression—*AtCOR47*, *AtRD29A*, *AtRD29B*, and *AtKIN1* genes after freezing stress in the transgenic *Arabidopsis*. The results indicate that the grapevine *CML21* gene acts as a positive regulator in the plant response to cold stress. The detected variety of *CML21* transcripts and their distinct transcriptional responses suggested that this expansion of mRNA variants could contribute to the diversity of grapevine adaptive reactions.

## 1. Introduction

Plants encode a remarkable variety of Ca^2+^-binding sensor proteins that are known to detect, decode, and transmit the changes in cellular Ca^2+^ concentrations to downstream events [1,2,3]. Calmodulin-like proteins (CMLs) constitute a large group of plant EF-hand containing Ca^2+^-binding proteins that have no enzymatic function but transmit information to downstream targets via Ca^2+^-dependent protein–protein interaction [4,5]. Therefore, CMLs were classified as sensor relay proteins [6,7]. While CMLs are closely related to calmodulin (CaM), a versatile calcium sensor relay protein in all eukaryotes, they are restricted only to higher plants and some protists [8]. CMLs considerably vary in their lengths containing only one to six predicted Ca^2+^-binding EF-hand motifs and lack any other known functional domains or intrinsic activities [4,5]. CaMs are more conserved proteins in structure harboring four EF-hands and encoded by a small gene family.

At present, CMLs remain the least studied family of plant Ca^2+^ sensor proteins. Much remains unknown about the molecular regulation and biological functions of individual CML family protein members. It is known that CMLs are implicated in plant responses to abiotic stresses [9], plant pathogen defense [10], growth and developmental processes [4,11]. For example, specific plant CMLs are known to regulate expression of stress/defense-related genes [12], the most abundant vacuolar Na+-proton exchanger, Na_+_/H_+_ exchanger 1 [13] or trichome branching [14]. All these molecular players and processes could contribute to plant adaptation to changing environment and unfavorable conditions. Constitutive expression of certain stress-responsive *CMLs* in transgenic plants revealed the involvement of the *CMLs* in plant responses to abiotic stresses as positive [15,16] and negative regulators [17,18]. Genome-wide expression analyses revealed that transcription activity of *CMLs* can be markedly affected in tea, apple, and grapevine plants by abiotic stimuli, including temperature stresses, high soil salinity, or drought [19,20,21,22]. Search for CML targets has shown that CMLs interact with diverse protein targets, e.g., transcription factors [23], DNA repair proteins [24] or ion transporters [13], which are known to be involved in plant adaptation to abiotic stress.

In a recent study, we identified and characterized the *CaM* and *CML* gene families in wild-growing grapevine *Vitis amurensis* Rupr. under the influence of abiotic stress conditions and found that most of the genes actively responded to abiotic stresses [22]. Wild-growing relatives of cultured plant species often exhibit higher tolerance to abiotic stresses. Therefore, regulatory genes of the wild plants represent an important source for improving abiotic stress tolerance of their cultivated counterparts. During previous work, we also found that several *CML* genes in *V. amurensis*, including *VaCML21*, undergo alternative splicing. Alternative splicing is the regulatory process where pre-mRNA fragments are rearranged by the spliceosomes generating several transcripts from one pre-mRNA molecule, which leads to the production of several protein isoforms. Alternative splicing is currently considered as a regulatory process expanding the genome capacity and protein diversity in response to the unfavorable environmental conditions [25]. 

Despite there being intron-rich *CMLs* and some other Ca*^2+^* sensors in the plant genome [3], the existing literature present scarce information on alternative splicing of plant Ca^2+^ sensors in general and do not present the evidence on alternative splicing of plant *CMLs*. To the best of our knowledge, there are only three investigations reporting on alternatively spliced plant Ca^2+^ sensor proteins, presented by three genes of Ca^2+^-dependent protein kinases (CDPKs or CPKs) [26,27,28]. CDPKs are the prominent Ca^2+^ sensors combining both Ca^2+^-binding and kinase domains [1,7]. Nishiyama et al. [26] reported that the liverwort *CDPK* gene has two almost identical and contiguous exons that were alternatively spliced into two mRNAs. These results indicated that the intron between the two alternative exons was not independently spliced out. Recently, Almadanim et al. [28] identified two alternatively spliced forms (alternative 3′ splice site selection) of rice *CPK17* gene that is required for a proper cold stress response. The *OsCPK17* splice transcript forms encoded truncated versions of the protein missing both the CDPK autoregulatory (activation) and Ca^2+^-binding domain due to a premature stop codon. The protein forms exhibited distinct functions and distributions. Previously, we found that the *CPK9* gene of *V. amurensis* undergoes unproductive alternative splicing mediated by canonical 5′GT and 3′AG splicing sites in *V. amurensis* to generate mRNA isoforms lacking key catalytic kinase subdomains [27]. In addition, Sanyal et al. [29] reported that the CBL-interacting protein kinase 3 (CIPK3) of *Arabidopsis* is regulated by alternative splicing. CIPKs are known to form functional complexes with calcineurin B-like proteins (CBLs), which are also important EF-hand containing Ca^2+^ sensors in plants [30]. 

Therefore, the purpose of the present study was to identify and describe the alternative spliced grapevine *CML21* forms and determine whether the spliced *CML21* variants play a role in grapevine responses to abiotic stress.

## 2. Results

### 2.1. VaCML21 Is Regulated by Alternative Splicing

**Figure 1:**
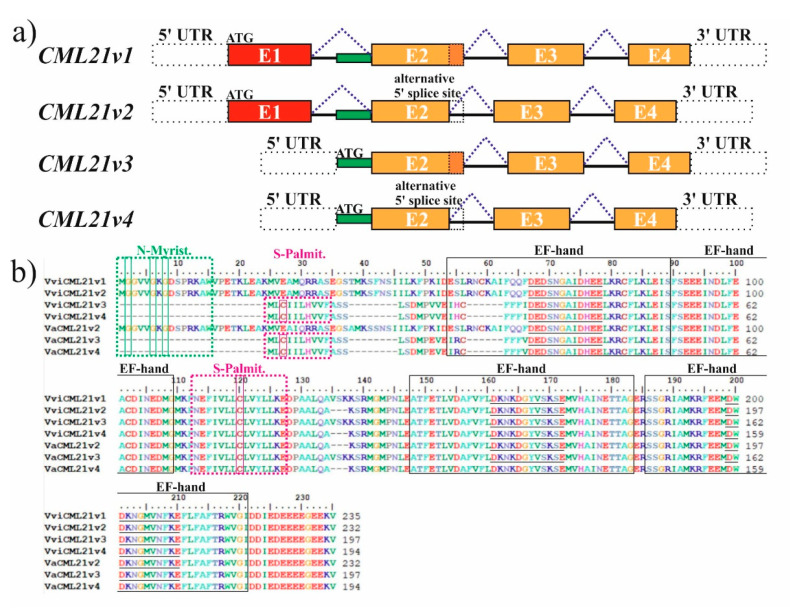
The structure representation and protein sequence analysis of *CML21* mRNA splice variants in grapevine. (**a**) Alternatively spliced *CML21* transcript variants of *Vitis amurensis* identified in this work (*VaCML21v2*, *VaCML21v3*, *VaCML21v4*) and *CML21* transcript variants of *Vitis vinifera* (*VviCML21v1*, *VviCML21v2*, *VviCML21v3*, *VviCML21v4*) predicted by genome sequence analysis and retrieved from the databases. Exons and introns are shown using boxes and lines, respectively, with white dashed boxes representing untranslated regions (UTRs). (**b**) Alignment and conserved motif analysis of *CML21* deduced proteins in *V. amurensis* and *V. vinifera*. The EF hands were predicted by PROSITE scan [31,32] and the lipid modification sites were predicted using GPS-Lipid [33,34]. Multiple sequence alignment was conducted using BioEdit software [35].

In addition to the *VviCML21v1* mRNA, which was predicted previously for *V. vinifera* [19], our analysis of the *V. vinifera CML* transcript sequences computationally predicted and retrieved from the CRIBI and NCBI databases made it possible to identify three additional *VviCML21* splice variants, i.e., *VviCML21v2*, *VviCML21v3*, and *VviCML21v4* (Figure 1; Table 1). Our transcript sequence analysis revealed that the transcription start sites differed between the *VviCML21v1/VviCML21v2* and *VviCML21v3/VviCML21v4* pairs. As a result, the *VviCML21v1* and *VviCML21v2* splice variants contained exon 1 in contrast to the *VviCML21v3* and *VviCMLv4*, where transcription was predicted to start later. This resulted in the loss of the exon 1 in *VviCML21v3* and *VviCMLv4* and a retention of the intron 1 partial sequence (Figure 1). Due to the partial intron retention, exon 2 was extended in the *VviCML21v3* and *VviCMLv4* splice variants (Figure 1). In addition, *VviCML21v1* and *VviCML21v3* transcript variants contained an identical insertion of nine nucleotides at the end of the exon 2 arising due to an alternative 5′ splice site selection (Figure 1). The boundaries of all putative introns in *CML21* contained the highly conserved splice site sequence patterns (both the 5′ donor GT and the 3′ acceptor AG splice sites), supporting the alternative splicing hypothesis. None of the four splice variants of *VviCML21* contained premature stop codons (no reading frame shift). 

Using RT-PCRs with specific primers designed for *VviCML21v1*, *VviCML21v2*, *VviCML21v3*, and *VviCML21v4* splice variants, we cloned and sequenced cDNAs containing full-length coding DNA sequences of *CML21* mRNAs from wild-growing *V. amurensis* using RNA samples isolated from the salt- *(VaCML21v2)* and cold-stressed *(VaCML21v3, VaCML21v4)* leaves of *V. amurensis*. The following sequencing analysis identified three splice variants of *CML21* in *V. amurensis*, including *VaCML21v2*, *VaCML21v3*, and *VaCML21v4* (Table 1). The transcripts *VaCML21v2*, *VaCML21v3*, and *VaCML21v4* shared a high nucleotide sequence identity with the *VviCML21v2*, *VviCML21v3*, and *VviCML21v4* (99%). We were not able to clone the *VaCML21v1*-like transcript variant using the RNA samples of *V. amurensis*. Table 1 shows a comparison of the cloned and sequenced *CML21* transcript forms of *V. amurensis* with those computationally predicted for *V. vinifera*. The deduced amino acid sequences of the cloned and sequenced *CML21s* of *V. amurensis* shared high identities with the homologous *CML21s* of *V. vinifera* (Table 1).

Like all plant CMLs, the deduced amino acid sequences of the *VviCML21* and *VaCML21* transcript variants did not contain functional domains other than EF-hands (Table 1). The main structural features and motifs of the deduced CML21s in *V. vinifera* and *V. amurensis* are depicted in Figure 1b. Absence of the exon 1 in the *VaCML21v3* and *VaCML21v4* transcript forms shortened the first EF hand, although the central 12 amino acid residues forming the central Ca^2+^ binding loop were not changed (Figure 1b). Furthermore, the computational prediction of post-translational modifications in the grapevine CML21s showed the presence of putative N-myristoylation sites in the CML21v1 and CML21v2 protein isoforms, while S-palmitoylation sites were detected in CML21v2, CML21v3, and CML21v4 (Table 1). The differences in the pattern of putative lipid modification sites were due to the removal of exon 1 and partial intron 1 retention (Figure 1). When comparing the deduced amino acid sequences of grapevine CML21s with the *Arabidopsis* CMLs, the grapevine CML21 shared the highest homology with AtCML21. However, to the best of our knowledge, the current literature does not present reports on the functions and expression pattern of AtCML21.

### 2.2. CML21 Expression Profile under Different Abiotic Stresses

**Figure 2:**
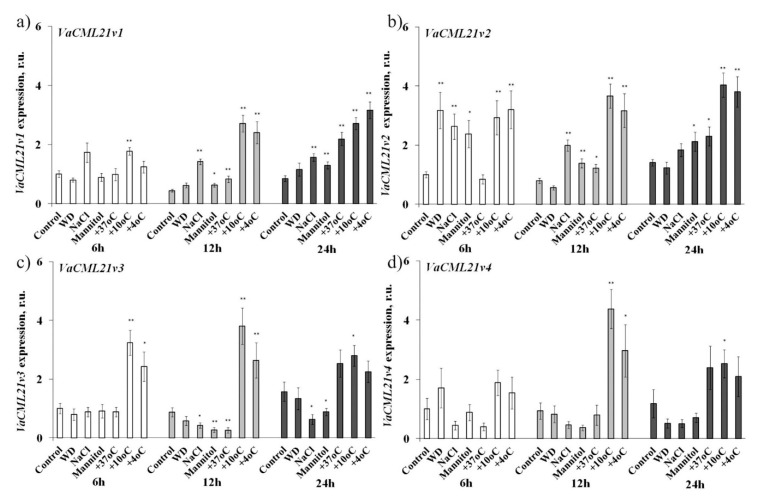
Expression analysis of *VaCML21v1* (**a**), *VaCML21v2* (**b**), *VaCML21v3* (**c**), and *VaCML21v4* (**d**) splice variants 6 h, 12 h, and 24 h post-treatment in the leaves of *V. amurensis* exposed to control (Control, filtered water, +25 °C), water-deficit (WD, cuttings laid on a paper towel, +25 °C), high salt (0.4 M NaCl, +25 °C), osmoticum (0.4 M mannitol, +25 °C), low temperature (+10 °C and +4 °C), and high temperature (+37 °C) abiotic stress conditions. The expression of the *CMLs* was profiled by quantitative real-time RT-PCR. The data are presented as the mean ± SE (three independent experiments). *, **—significantly different from the Control for each time point at *p* ≤ 0.05 and 0.01 according to Student’s *t*-test.

The transcript abundance of the *VaCML21v1, VaCML21v2*, *VaCML21v3*, and *VaCML21v4* splice variants were analyzed under desiccation, high salinity, high mannitol, cold and heat stresses in the cuttings of wild-growing grape *V. amurensis* by quantitative real-time RT-PCR (qRT-PCR) (Figure 2). The levels of all four *VaCML21* transcript forms were highly up-regulated under both +10 °C and +4 °C cold stress treatments at a minimum of two treatment intervals (Figure 2). Among the four splice variants, the level of *VaCML21v4* showed a lower cold stress responsiveness being cold-regulated only 12 h and 24 h post-treatment. At the same time, the transcript pairs *VaCML21v1/v2* and *VaCML21v3/v4* exhibited distinct inducibility in respect of other abiotic stresses (Figure 2). While the structurally similar pair *VaCML21v1* and *VaCML21v2* was activated in response to salt, desiccation, mannitol, and heat stress, the pair of *VaCML21v3* and *VaCML21v4* either did not respond to these stresses or were even down-regulated under the treatments (Figure 2). 

**Figure 3:**
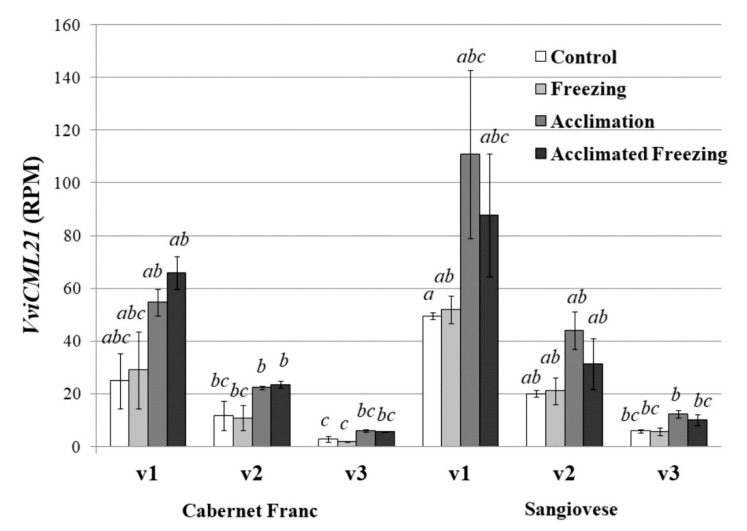
Expression levels of the *CML21v1*, *CML21v2*, and *CML21v3* splice variants in *Vitis vinifera* in the most-damaged cultivar “Cabernet Franc” and the least-damaged cultivar “Sangiovese” cultivated under low-temperature stress conditions. The RNAseq libraries were obtained by Londo et al. [37] and analyzed in the present study for *VviCML21* abundance. The *V. vinifera* plants were exposed to control treatment, freezing (gradual freezing to −3 °C for 45 min), acclimation or chilling (+4 °C for 48 h), and acclimated freezing (+4 °C for 48 h and −3 °C for 45 min). RPM—reads per million mapped reads. The data are presented as the mean ± SE. Means followed by the *same letter* were not different using one-way analysis of variance (ANOVA), followed by the Tukey HSD multiple comparison test. A value of *p* ≤ 0.05 was considered significant.

In the present work, we downloaded the RNAseq libraries of cold-treated *V. vinifera* by Londo et al. [37] from the Sequence Read Archive (SRA) and analyzed the content of the individual *VviCML21v1*, *VviCML21v2*, and *VviCML21v3* transcripts under acclimation (chilling) and/or freezing stress (Figure 3). We did not find *VviCML21v4* in the analyzed RNAseq libraries. Two different *V. vinifera* cultivars were chosen for this analysis: the most-damaged cultivar “Cabernet Franc” and the least-damaged cultivar “Sangiovese” (Figure 3). The data analysis revealed that the chilling acclimation, i.e., chilling stress, but not freezing itself, increased the transcript abundance of *VviCML21v1*, *VviCML21v2*, and *VviCML21v3*. Freezing without the chilling pretreatment did not have an effect on *VaCML21* transcript abundance. Notably, transcript abundance of *VviCML21v1*, *VviCML21v2*, and *VviCML21v3* was higher in the cold-resistant cultivar “Sangiovese” than that in the cold-sensitive cultivar “Cabernet Franc” by 1.4–3.1 times. In addition, we noted that the *VviCML21v3* splice variant was produced at a much lower level than *VviCML21v1* and *VviCML21v2*. 

### 2.3. Genetic Plant Transformation with the VaCML21v2 and VaCML21v4 Splice Variants and Selection of the Transformants

**Figure 4:**
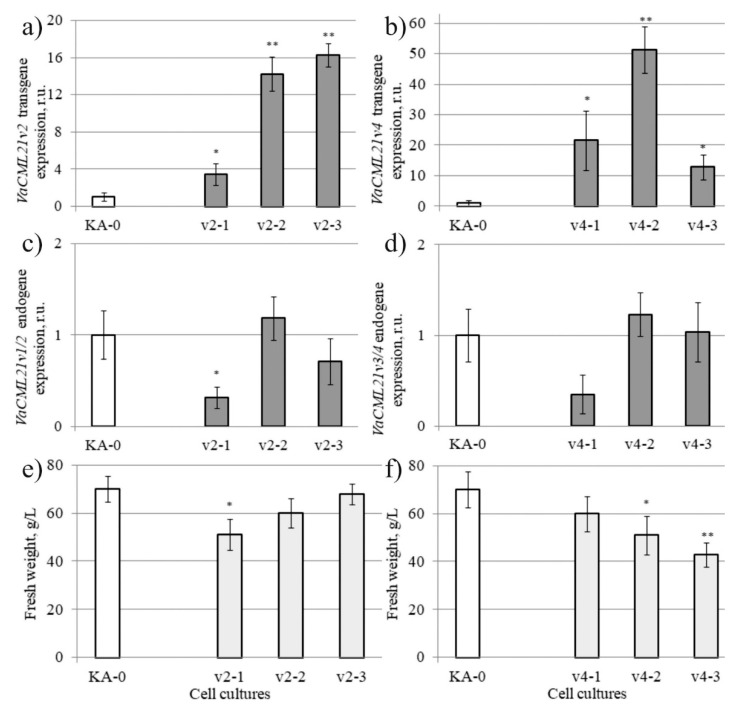
Characterization of the grapevine callus cell cultures transformed with the *VaCML21v2* and *VaCMLv4* splice variants. (**a**,**b**) Quantification of the *VaCML21v2* and *VaCML21v4* transgene mRNAs; (**c**,**d**) Quantification of the endogenous *VaCML21v1/v2* and *VaCML21v3/v4* mRNAs. (**e**,**f**) Fresh biomass accumulation in the *VaCML21v2*-transgenic and *VaCML21v4*-transgenic cell cultures after 30-day cultivation. v2-1, v2-2, v2-3—*VaCML21v2*-transformed calli; v4-1, v4-2, v4-3—*VaCML21v4*-transformed calli. KA-0—control cell culture transformed with the “empty” vector. The data are presented as the mean ± SE (two independent experiments). ^*^ *p* ≤ 0.05; ^**^ *p* ≤ 0.01 versus values of *VaCML21* expression in the empty vector-transformed KA-0 cell culture according to Student’s *t*-test.

To gain further insight into the involvement of *CML21* into the grapevine response to abiotic stresses, we transferred the 35s::*VaCML21v2* and 35s::*VaCML21v4* splice variants to cell suspension of *V. amurensis* and established independent transgenic callus cell lines. qRT-PCR revealed that the *VaCML21v2*-transformed (v2-1, v2-2, v2-3) and *VaCML21v4*-transformed (v4-1, v4-2, v4-3) callus cell cultures actively expressed the *VaCML21v2* and *VaCML21v4* transgenes (Figure 4a,b). qRT-PCR revealed that the levels of endogenous *VaCML21v1/v2* and *VaCML21v3/v4* mRNAs were not sufficiently changed, except for those in v2-1 cell line (Figure 4c,d). Under standard cultivation conditions, the v2-1, v4-2, and v4-3 cell lines accumulated biomass at a lower level than the KA0 control during the 30 d of cultivation (Figure 4e,f).

**Figure 5:**
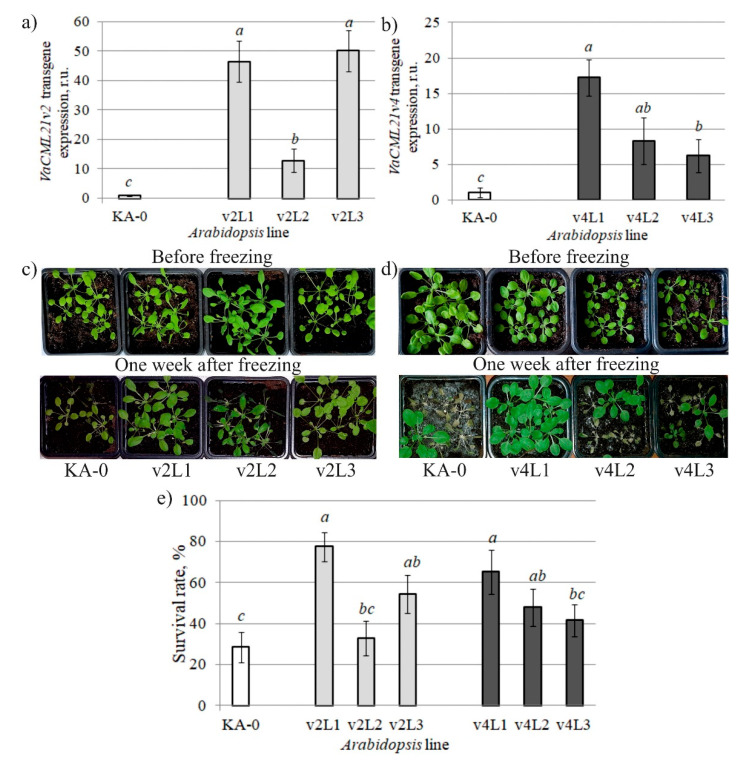
Characterization and responses to freezing of vector control (KA-0), *VaCML21v2-* (v2L1, v2L2, v2L3) and *VaCML21v4*-transgenic (v4L1, v4L2, v4L3) *Arabidopsis*. (**a**,**b**) Quantification of the *VaCML21v2* and *VaCML21v4* mRNAs in *A. thaliana* performed by qRT-PCR. (**c**,**d**) 4-week-old KA-0 and *VaCML21*-transgenic plants were stressed at −10 °C for 45 min and then cultured at +4 °C for 1 h for recovery. Photographs of representative seedlings were taken after 7 d of recovery. (**e**) Survival rates determined as the number of visibly green plants 7 d after freezing. Values are the mean ± SE. Six plants of each line were used in each of ten experiments. Means followed by the *same letter* were not different using one-way analysis of variance (ANOVA), followed by the Tukey HSD multiple comparison test. A value of *p* ≤ 0.05 was considered as significant.

Then, the overexpression construct of *VaCML21* or empty vector was transformed in *Arabidopsis* for generating the *VaCML21v2*-transgenic (v2L1, v2L2, v2L3), *VaCML21v4-*transgenic (v4L1, v4L2, v4L3), and control (KA-0) *A. thaliana* plant lines. The transgenic plant lines showed no morphological aberrations through the T_4_ generation. Quantification of the *VaCML21v2* and *VaCML21v4* mRNAs in the transgenic *A. thaliana* was performed by qRT-PCR (Figure 5a,b). As shown in Figure 5a,b, the transgenic *35S::VaCML21v2* and *35S::VaCML21v4 A. thaliana* displayed different levels of *VaCML21v2* and *VaCML21v4* expression with the highest level of transgene mRNAs being detected in the v2L1, v2L3, and v4L1 lines. Expression of the *VaCML21v2* transgene in v2L1 and v2L3 was considerably higher than that observed in v2L2 (by 3.6–3.9 times), while expression of *VaCML21v4* transgene in v4L1 markedly exceeded the transgene levels in v4L2 and v4L3 (by 2.1–2.8 times). 

### 2.4. Stress Tolerance of VaCML21-Overexpressing V. amurensis Cell Cultures and A. thaliana Plants

**Figure 6:**
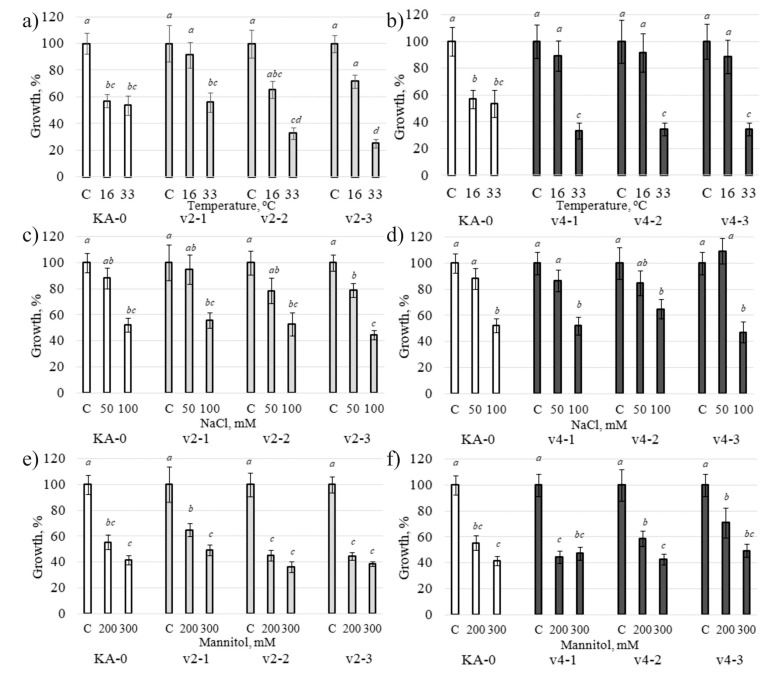
Influence of temperature (**a**,**b**), salt (**c**,**d**) and osmotic (**e**,**f**) stresses on the fresh biomass accumulation in the transgenic grapevine callus cell lines overexpressing the *VaCML21v2* and *VaCML21v4* splice variants. The average growth rates were assessed after 30 days of cultivation in the dark under the control (24 °C), cold stress (+16 °C), heat stress (+37 °C), salt stress (NaCl 50 and 100 mM), and high-mannitol (200 and 300 mM) conditions. v2-1, v2-2, v2-3—*VaCML21v2*-transformed calli; v4-1, v4-2, v4-3—*VaCML21v4*-transformed calli. KA-0—control cell culture transformed with the “empty” vector. The data are presented as the mean ± SE (two independent experiments with ten replicates each). Means followed by the *same letter* were not different using one-way analysis of variance (ANOVA), followed by the Tukey HSD multiple comparison test. A value of *p* ≤ 0.05 was considered as significant.

To assess the effect of cold and heat stress conditions on the cell culture biomass accumulation, we cultivated the *VaCML21v2*- and *VaCML21v4*-transgenic calli at 16 °C and 33 °C for 30 days (Figure 6a,b). To assess the effect of high salinity and mannitol-induced osmotic stress, the calli were also cultivated in the presence of NaCl or mannitol for 30 days (Figure 6c–f). Under high mannitol and salt stress conditions, the growth of the v2-1,2,3 and v4-1,2,3 cell lines was reduced to approximately the same level as the growth of the control KA-0 calli. However, the *VaCML21v2-* and *VaCML21v4*-transformed calli showed a higher biomass accumulation in response to cold stress in comparison with the growth of the KA-0 control (Figure 6a,b). Under cold stress conditions, growth of the v2-1,2,3 and v4-1,2,3 calli was reduced 1.1–1.5-fold and 1.1–1.2-fold, respectively, while the growth of KA-0 was reduced 1.8-fold. Under heat stress conditions, most of the *VaCML21*-transgenic lines exhibited a lowered biomass accumulation in comparison with that of KA0 control.

The stress tolerance assays of the transgenic *A. thaliana* showed that the survival rates of the *35S::VaCML21* lines were higher than the survival rates of the KA-0 control plants (Figure 5c–e). The increase in cold stress tolerance was statistically significant only for those two transgenic lines (v2L1, v2L3, v4L1, v4L2) that expressed the *VaCML21v2* and *VaCML21v4* splice variants at a higher level (Figure 5a,b,d). 

### 2.5. Transformation with VaCML21 Enhanced Transcription of Cold-Inducible Regulatory Genes in Stressed Arabidopsis

**Figure 7:**
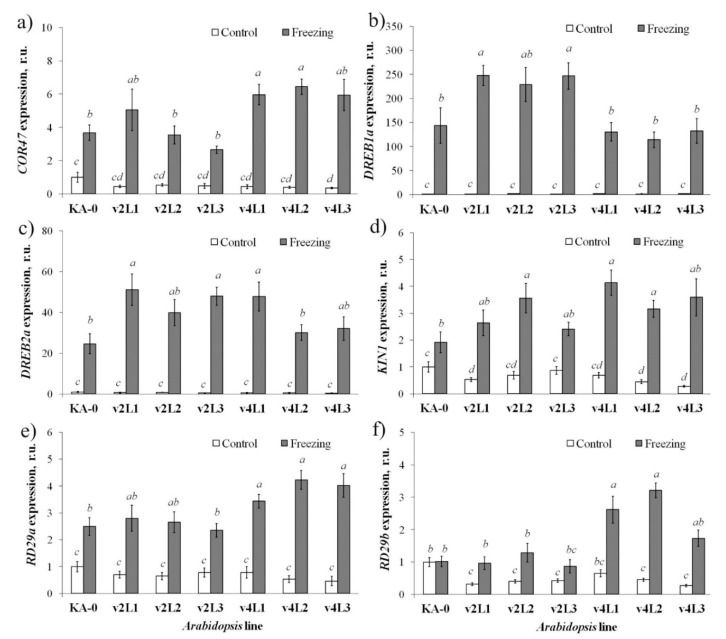
The expression of stress-responsive genes *AtCOR47* (**a**), *AtDREB1A* (**b**), *AtDREB2A* (**c**), *AtKIN1* (**d**), *AtRD29A* (**e**), and *AtRD29B* (**f**) in transgenic *Arabidopsis* transformed with the *VaCML21v2* and *VaCML21v4* splice variants in response to freezing stress. The 4-week-old control KA-0, 35S::*VaCML21v2* (lines v2L1, v2L2, and v2L3), and 35S::*VaCML21v4* (lines v4L1, v4L2, and v4L3) plants were exposed to control conditions (at 22 °C) or freezing (at −10 °C for 45 min and then cultured at +4 °C for 1 h for recovery). Total RNA was extracted from plants just before freezing (white bars) and 1 h after freezing (grey bars). The data are presented as the mean ± SE (two independent experiments). Means followed by the *same letter* were not different using one-way analysis of variance (ANOVA), followed by the Tukey HSD multiple comparison test. A value of *p* ≤ 0.05 was considered significant.

To further evaluate the involvement of *VaCML21* to cold stress response, we analyzed expression of nine cold-inducible genes, including *AtCOR47*, *AtRD29A*, *AtRD29B*, *AtDREB1A*, *AtDREB2A*, *AtKIN1*, *AtLEA*, *AtCBF1*, and *AtRAB18*, which are known to function in plant cold stress protection and signaling, in the control and *VaCML21*-transgenic plants after freezing (Figure 7). The data revealed that the expression of the cold stress-associated genes did not differ between the KA-0 control and *VaCML21*-transgenic lines under normal conditions, except for lowered AtRD29B expression. However, in response to freezing, expression of six genes *AtCOR47*, *AtRD29A*, *AtRD29B*, *AtDREB1A*, *AtDREB2A*, *AtKIN1* was up-regulated either in the *VaCML21v2*- or *VaCML21v4*-transgenic *Arabidopsis* lines to a considerably higher degree than in the control KA-0 plant line of *A. thaliana* (Figure 7). Overexpression of *VaCML21v2* led to enhanced induction of *AtDREB1A* and *AtDREB2A* in response to freezing in at least two lines (Figure 7b,c), while overexpression of *VaCML21v4*—*AtCOR47*, *AtKIN1*, *AtRD29A*, and *AtRD29B* (Figure 7a,d,e,f) Transcription abundance of *AtLEA*, *AtCBF1*, and *AtRAB18* was not essentially changed under either the control conditions or after freezing in all plant lines (data not shown).

## 3. Discussion

The diverse and unique plant *CML* subfamily has been reported to serve as a set of Ca^2+^ sensor relay proteins with important functions in plant responses to environmental cues and developmental processes [6,7]. The presence of several introns in some plant *CMLs* makes the genes a target of alternative splicing, a process where generation of several mRNA splice variants could contribute to the diversity of plant reactions and stress adaptation. Despite there being intron-rich *CMLs* and some other Ca*^2+^* sensors in the plant genome [3], the existent literature present little information about alternative splicing of Ca^2+^ protein sensors in plants. This study showed that the grapevine *CML21* gene produced four splice variants arising from using an alternative transcription start site and a partial intron retention events combined with alternative splice site selection. The modifications affected CML21 structure, including the type of putative lipid modification sites and structure of the first EF-hands. These changes could potentially change localization and Ca^2+-^ binding properties of CML21. 

The present analysis revealed that all four splice variants of *V. amurensis CML21* were highly activated in response to cold stress. The data on *V. amurensis* were in accordance with the chilling inducibility of the *V. vinifera CML21*. At the same time, *VaCML21v1* and *VaCML21v2* (but not *VaCML21v3* and *VaCMLv4*) variants were highly activated in response to several other abiotic stress treatments, including desiccation, high mannitol, salinity, and heat stress. Overexpression of the *VaCML21v2* and *VaCML21v4* splice variants in the callus cultures of *V. amurensis* resulted in a higher biomass accumulation of the *CML21v2-* and *CML21v4*-transgenic cell lines during cultivation under lowered temperature, supporting the positive role of *CML21* in cold stress adaptation. Furthermore, heterologous overexpression of the grapevine *CML21v2* and *CML21v4* splice variants in *Arabidopsis* led to improved survival rates of the transgenic plant lines after freezing and resulted in an enhanced transcriptional responsiveness of the cold stress-inducible genes *AtCOR47*, *AtRD29A*, *AtRD29B*, *AtDREB1A*, *AtDREB2A*, and *AtKIN1* to freezing stress. The transcription factors DRE-BINDING PROTEIN 1A and 2A (DREB2A and DREB1A) have been reported to regulate dehydration-responsive element (DRE)-mediated transcription of target genes under cold stress, dehydration, and high salinity [38,39]. The expression of *COR* genes has been shown to be critical in plants for cold stress acclimation and tolerance [40]. The *COR47*, *RD29A*, *RD29B*, *LEA*, and some other *COR* genes known as group 2 LEA (LEA II) proteins, encode dehydrins, which are cold-inducible proteins that are supposed to sustain membrane stabilization and prevent protein aggregation [41]. The cold-inducible protein AtKIN1 has been suggested to mediate both cold and osmotic stress responses and possesses similarities with antifreeze proteins [42]. 

Thus, the *VaCML21v2* and *VaCML21v4* splice variants exhibited not only distinct expression patterns in response to abiotic stress treatments of *V. amurensis*, but also induced different cold stress-protective genes in response to freezing in *A. thaliana*. This indicates a distinct molecular mechanism for the *VaCML21v1/v2-* and *VaCML21v3/v4-*mediated regulation of cold stress response in grapevine. The mRNA transcript diversity of some Ca^2+^ sensor proteins and their distinct transcriptional properties detected in this and other works suggested that this splicing-induced expansion of mRNA variants could contribute to the diversity of plant adaptive reactions to unfavorable environmental conditions. 

## 4. Materials and Methods

### 4.1. Plant Materials and Growth Conditions

For the abiotic stress treatments, we used young vines of wild-growing grapevine *V. amurensis* Rupr. (Vitaceae) sampled from a non-protected natural population near Vladivostok, Russia (Akademgorodok, the southern Primorsky region of the Russian Far East, longitude 43.2242327 and latitude 131.99112300) and identified at the Botany Department of the Federal Scientific Center of the Biodiversity FEBRAS. The freshly harvested *V. amurensis* vines were divided into cuttings (excised young stems 7–8 cm long with one healthy leaf) that were placed in individual beakers and used for the stress treatments. The cuttings were placed into the filtered water at 25 °C immediately after cutting. Then, the cuttings were acclimated to the “non-stress” condition for 30 min before they were treated with the stress treatments. For the control non-stress treatment, the *V. amurensis* cuttings were placed in filtered water at 25 °C. To induce water deficit stress, the cuttings were laid on a paper towel at 25 °C. To induce osmotic stress, the cuttings were placed in 400 mM NaCl (Himreaktivsnab, Ufa, Russia) and 400 mM D-mannitol (AppliChem, Darmstadt, Germani) solutions at 25 °C. To apply cold and heat stress, the *V. amurensis* cuttings were placed in filtered water in the growth chamber (Sanyo MLR-352, Panasonic, Osaka, Japan) at +4 °C, +10 °C, and +37 °C. The *V. amurensis* cuttings were grown under a 16/8 h light/dark photoperiod. We used similar experimental design and employed the same NaCl and mannitol concentrations as Chung et al. [43] and Dubrovina et al. [44] for studying calcium-dependent protein kinase gene expression in *Capsicum annuum* and *V. amurensis*. The experiments were repeated three times for each stress treatment time and for the control treatment.

Plants (*Arabidopsis thaliana* ecotype Columbia L., stored by our lab) were grown in pots filled with commercially available rich soil in a controlled environmental chamber at 22 °C (Sanyo MLR-352, Panasonic, Japan) kept on a 16/8 h day/night cycle at a light intensity of ~120 μmol m^−2^ s^−1^.

### 4.2. Isolation and Sequencing of VaCML21 Spliced Transcript Variants

Full-length cDNA coding sequences of *VaCML21v2*, *VaCML21v3*, and *VaCML21v4* were amplified using RNA samples extracted from stressed leaves of *V. amurensis,* the high-fidelity Tersus polymerase (Evrogen, Moscow, Russia) and primers designed to the predicted coding sequences of *VvCML21* mRNAs of *V. vinifera* PN20024 genotype (V2 mRNA prediction) retrieved from the Grapevine Genome Database hosted at Centro Di Ricerca Interdipartimentale Per Le Biotecnologie Innovative (CRIBI) Biotech Centre (http://genomes.cribi.unipd.it/grape; Table 2). The RT-PCR products were subcloned into pJET1.2/blunt and sequenced as described previously [22]. The VaCML amino acid sequences were predicted using the Gene Runner program (http://www.generunner.net). We performed a domain analysis by PROSITE scan ([31]; http://prosite.expasy.org), calculated Compute pI/Mw tool (http://web.expasy.org/compute_pi) and prediction of myristoylation and palmitoylation motif numbers with GPS-Lipid ([32]; http://lipid.biocuckoo.org/webserver.php). Multiple sequence alignments were done with the BioEdit 7.0.8 program [35]. The full-length coding mRNA sequences of *VaCML21* and *VviCML21* splice variants are given in Table S1.

### 4.3. Generation of Transgenic Grapevine Cell Cultures

The overexpression constructs of *VaCML21* (pZP-RCS2-*VaCML21v2*-*nptII*; pZP-RCS2- *VaCML21v4*-*nptII*) or empty vector (pZP-RCS2-*nptII*) were obtained as described [45]. Briefly, the full-length cDNA of *VaCML21v2* and *VaCML21v4* were amplified by PCR using the primers listed in Table 2 from pJET1.2. The full-length cDNAs of *VaCML21v2* and *VaCML21v4* were cloned into the pZP-RCS2-*nptII* vector [46] under the control of the double cauliflower mosaic virus (CaMV 35S) promoter. The pZP-RCS2-*nptII* construction also carried the *nptII* gene under the control of the double CaMV 35S promoter. Plasmid DNA samples (pSAT1 and pZP-RCS2-*nptII*) were kindly provided by Professor Alexander Krichevsky (State University of New York, Stony Brook, USA). The overexpression constructs were introduced into the *Agrobacterium tumefaciens* strain GV3101:pMP90 and transformed into the suspension culture V7 of *V. amurensis* by co-cultivation as described [47]. Briefly, *A. tumefaciens* strains bearing pZP-RCS2-*nptII* or pZP-RCS2-*VaCML21v2/v4*-*nptII* constructs were inoculated in multiple separate flasks with cell suspensions of *V. amurensis* to establish the independently transformed KA-0 (empty vector), *VaCML21v2*-transgenic (v2-1, v2-2, v2-3), and *VaCML21v4*-transgenic (v4-1, v4-2, v4-3) callus cell cultures. The KA-0 cell culture was used as a control in all further experiments. The V7 callus culture were established in 2017 from young stems of the mature *V. amurensis* plants as described [48]. After the transformation, the calli were cultivated for a 4-month period in the presence of 10–20 mg/L of Km to select transgenic cells and for a 6-month period in the presence of 250 mg/L of Cf to suppress the bacteria. Transgenic callus cell cultures were selected as described [47]. Transgenic callus cell cultures were selected and confirmed by PCR as described [45]. To analyze the level of exogenous *VaCML21v2* and *VaCML21v4* (transgenes), we used primers designed to the CaMV 35S promoter and the 5’ end of the *VaCPK21* protein coding region (Table 2). To analyze the level of endogenous *VaCML21* splice variants, we used primers designed to the 5′ UTR and the 5′ end of the *VaCPK21* protein coding region (Table 2).

### 4.4. Generation of Transgenic Arabidopsis Plants

To create *A. thaliana* lines overexpressing the *VaCML21v2* and *VaCML21v4* splice variants, we used the same plasmid construction as for overexpression of *VaCPK21* in cell cultures of *V. amurensis* (described above). The overexpression construct of *VaCPK21* or empty was introduced into the *A. tumefaciens* strain GV3101::pMP90 and transformed by floral dip method into wild-type *A. thaliana* [49]. Transgenic plants were selected and confirmed by PCR as described [50] using primers designed for the CaMV 35S promoter in the pSAT1 vector and the 5′ end of the *VaCPK21* protein coding region (Table 2). The PCR products were verified by DNA sequencing. The transgenic lines used in this study were homozygous plants with a single copy insertion. Six representative independent T_3_ homozygous lines (v2L1, v2L2, v2L3, v4L1, v4L2, v4L3) with high mRNA levels of the *VaCML21v2* and *VaCML21v4* transgenes were chosen for further analysis. We also selected a T_3_ homozygous KA-0 line of *A. thaliana* transformed with the empty vector (pZP-RCS2-*npt*II).

### 4.5. RNA Isolation and cDNA Synthesis

Total RNA isolation was performed using the cetyltrimethylammonium bromide-based extraction [51]. Complementary DNAs were synthesized using 1.5 µg of total RNA by the MMLV RT Kit (Evrogen, Moscow, Russia). The reactions were performed in 20 µL aliquots of the reaction mixture, which contained the first strand buffer, 2 µL of dNTP mix (10 mM each), 2 µL of oligo-(dT)15 primer (20 µM), and 2 µL of MMLV reverse transcriptase (100 u/µL), at 37 °C for 1.5 h. The 1 µL samples of reverse transcription products were then amplified by PCR and verified on the absence for DNA contamination using primers listed in Table 2.

### 4.6. Gene Expression Analysis by qRT-PCR

The qRT-PCRs were performed with EvaGreen Real-Time PCR (Biotium, Hayward, CA, USA) as described in [52,53], using two internal controls (*VaActin1*, *VaGAPDH, AtGAPDH, AtEF1a*). The expression was calculated by the 2^−ΔΔCT^ method [54]. All GenBank accession numbers and primers are listed in Table 2. The specific primers for qRT-PCR were designed for the regions specific to each *CML21* splice variant (Table 2). The qRT-PCR data shown were obtained from at least two independent experiments and are averages of eight technical replicates for each independent experiment (four qPCR reactions normalized to *Actin* and four qPCR reactions normalized to *GAPDH* expression for each independent experiment).

### 4.7. V. vinifera RNAseq Library Analsis

The sequences of the *VviCML21* four transcript variants were combined with the grapevine genome file (https://www.ncbi.nlm.nih.gov/genome/401?genome_assembly_id=214125). Bowtie2 (bowtie2-build genome_name.fna index_db) was used to construct index files Bowtie2 for the *V. vinifera* genome. Recently, Londo et al. 2018 [37] produced Illumina RNAseq libraries of to analyze the broad transcriptional response of *V. vinifera* to low temperature stress. The RNAseq libraries [37] were downloaded from the Sequence Read Archive (SRA) data available through NCBI (accession: PRJNA402079). The RNAseq library numbers and cultivation conditions are listed in Table 3. The SRA files in fastq format were aligned to the *V. vinifera* indexed genome using Bowtie2 (bowtie2-q -x index_db -U SAR_file.fastq -S output_file.sam --un-gz 1.un.fq.gz -N 1 –local). Samtools was used to convert the SAM files to BAM files (samtools view file.sam –b –o file.bam) and for further sortingBAM files (samtools sort –l 9 file.bam –o file.sorted.bam). The command “samtools view –c” (samtools view –c file.sorted.bam “sample number ID”) was used to quantitate the number of primary aligned reads in each sample for each treatment. All bioinformatics assays were performed “blind” and were-based solely on sample number ID. The resulting number of reads was recalculated per million reads of the SRA file.

### 4.8. Abiotic Stress Treatments of Transgenic Cell Cultures and Plants

The *V. amurensis* calli were cultivated in 150 × 16 mm test tubes containing 9 mL of agarized Murashige and Skoog (MS) modified W_B/A_ medium [55] supplemented with 0.5 mg/L 6-benzylaminopurine and 2.0 mg/L α-naphthaleneacetic acid. Growth analysis and abiotic stress treatments of transgenic callus cell cultures were conducted as described [50,56]. Briefly, salt treatment was applied by adding 50 and 100 mM of NaCl to the W_B/A_ culture media. Mannitol treatment was applied by adding 200 and 300 mM of mannitol to the medium. Cold and heat treatments were performed by culturing the transgenic cells at 16 °C and 33 °C in a growth chamber (TSO-1/80 SPU, SKTB, Smolensk, Russia). The average growth rates were assessed after 30 days of cultivation under the control and stress conditions in the dark at 24–25 °C. The data were obtained from two independent experiments with 10 replicates each.

The plants of *A. thaliana* were subjected to freezing as described [50,52]. Briefly, the sterilized transgenic seeds of *Arabidopsis* were germinated on plates and the seven-day-old seedlings were transferred to commercially available rich well-watered soil in a controlled environmental chamber at standard conditions. For the low-temperature stress, normally cultured four-week-old *A. thaliana* plants were stressed in a −10 °C freezer for 45 min and then cultured at +4 °C for 1 h before RNA isolation. Six plants of each line were used in each of ten experiments.

### 4.9. Statistical Analysis

The data are presented as mean ± standard error (SE) and were evaluated by Student’s *t*-test (expression analysis for the grapevine cuttings and callus cultures) or one-way analysis of variance (ANOVA) (all other analyses), followed by the Tukey HSD multiple comparison test. A value of *p* < 0.05 was considered significant. 

## Figures and Tables

**Table 1:** Sequence characteristics of the *CML21* mRNAs and their corresponding proteins in *Vitis amurensis* and *Vitis vinifera.*

*Vitis amurensis* (cDNA Clone)			No. of EF Hands	Predicted Lipid Modification Sites (Position)	MW (kDA)	*Vitis vinifera* (mRNA Prediction)			No. of EF Hands	Predicted Lipid Modification Sites (Position)	MW (kDA)
Transcript Variant	ID (NCBI GenBank)	CDS (Amino Acids)				Transcript Variant	ID	CDS (Amino Acids)			
nd	nd	nd	nd	nd	nd	*VviCML21v.1*	VIT_219s0015g01200.1	708 (235)	4	N-Myrist(2,6,8)	26.9
*VaCML21v.2*	MN540599	699 (232)	4	N-Myrist(2,6,8) N-Palmit(120)	26.4	*VviCML21v.2*	VIT_219s0015g01200.2	699 (232)	4	N-Myrist(2,6,8) N-Palmit(120)	26.5
*VaCML21v.3*	MW033207	594 (197)	4	N-Palmit(3)	22.7	*VviCML21v.3*	VIT_219s0015g01200.7	594 (197)	4	N-Palmit(3)	22.7
*VaCML21v.4*	MW033208	585 (194)	4	N-Palmit(3,82)	22.4	*VviCML21v.4*	VIT_219s0015g01200.8	585 (194)	4	N-Palmit(3,82)	22.4

Note: No. of EF hands—the number of EF hands predicted by PROSITE scan [31,32]; N-Myrist and N-Palmit—the myristoylation and palmitoylation motifs identified with GPS-Lipid [33,34]; CDS—coding mRNA sequences (bp); MW (kDa)—molecular mass calculated using the Compute pI/Mw tool [36].

**Table 2:** Primers used for amplification of grapevine *CML21* cDNAs and the house-keeping genes.

Transcript Name (GenBankID)	Primers, 5′–3′
Cloning and sequencing full-length cDNA coding sequences of *VaCML21*, 5′–3′	
*CML21v1/v2* (no sequence/MN540599)	F1:ATGGGAGGCGTGGTGGG R:TCAAACTTTCTCTTCACCTTC
*CML21v3/v4* (MW033207/ MW033208)	F2:ATGCTGTGTATCATCCTTCATG R:TCAAACTTTCTCTTCACCTTC
Cloning *VaCML21v2 and VaCML21v4* for plant transformation by the *Bgl*II and *Sal*I sites (underlined), 5’–3’	
*VaCML21v2* (MN540599)	F:GCTCAGATCTATGGGAGGCGTGGTGGGAAAA R:TCGAGTCGACTTAAACTTTCTCTTCACCTTC
*VaCML21v4* (MW033208)	F:GCTCAGATCTATGCTGTGTATCATCCTTCAT R:TCGAGTCGACTCAAACTTTCTCTTCACCTT
Primers for real-time PCR, 5′–3′	
*VaCML21v1* (no sequence)	GAACTGCAAAGCTATTTTTCAGCA CCCATCCGTGATTTTTTGGATAC
*VaCML21v2* (MN540599)	GAACTGCAAAGCTATTTTTCAGCA ATCCGTGATTTGGCCTGAAGG
*VaCML21v3* (MW033207)	TGCCAGAGGTTGAAATTCGTTGTT CCCATCCGTGATTTTTTGGATAC
*VaCML21v4* (MW033208)	TGCCAGAGGTTGAAATTCGTTGTT ATCCGTGATTTGGCCTGAAGG
*VaActin1* (DQ517935)	GTATTGTGCTGGATTCTGGTGAT AGCAAGGTCAAGACGAAGGATAG
*VaGAPDH* (XM_002263109)	CACTGAAGATGATGTTGTTTCC GCTATTCCAGCCTTGGCAT
*AtGAPDH* (NM_111283.4)	TTGGTGACAACAGGTCAAGCA AAACTTGTCGCTCAATGCAAT
*AtEF1a* (XM_002864638)	TGAGCACGCTCTTCTTGCTTTCA GGTGGTGGCATCCATCTTGTTACA
Primers for cDNA check-up on DNA contamination, 5′–3′	
*VaActin1* (DQ517935)	TTGCCATTCAGGCTGTTCTTTCT AGGAGCTGCTCTTTGCAGTTTCC
*AtActin1* (NM_112764)	GATTCAGATGCCCAGAAGTC TCTGTGAACGATTCCTGGA
Primers for qRT-PCR quantification of the *VaCML21v2* and *VaCML21v4* transgene and endogene mRNAs in transformed plant cells, 5′–3′	
*VaCML21v2* transgene (MN540599)	CAAGCATTCTACTTCTATTG ACCATTTTGGCCTCAAGC
*VaCML21v1/v2* endogene (no sequence/MN540599)	CATACTTAGCACTTGTCCCTTTTCC ACCATTTTGGCCTCAAGC
*VaCML21v4* transgene (MW033208)	CAAGCATTCTACTTCTATTG TCTTCATAGATGAGGATTCAAATG
*VaCML21v3/v4* endogene (MW033207/ MW033208)	TGAACCAGTTTTCATTTTAATTCAACGTG TCTTCATAGATGAGGATTCAAATG

**Table 3:** RNAseq libraries of *Vitis vinifera* cultivated under cold stress [37].

Sangiovese		Cabernet Franc		Cultivation Conditions ^a^
SRR6026721	SRR6026711	SRR6026737	SRR6026699	Control
SRR6026722	SRR6026712	SRR6026736	SRR6026700	Freezing (−3) for 45 min
SRR6026716	SRR6026735	SRR6026738	SRR6026698	Chilling stress (+4) for 48 h
SRR6026715	SRR6026734	SRR6026739	SRR6026697	Acclimated freezing (+4 → −3)

^a^ A detailed description of the cultivation conditions was presented in [37].

## Supplementary Materials

Supplementary materials can be found at https://www.mdpi.com/1422-0067/21/21/7939/s1. Table S1. The nucleotide sequences of *CML21* splice variants of *Vitis vinifera* and *Vitis amurensis*.
