# Supplementary material for: The Grapevine Calmodulin-Like Protein Gene CML21 Is Regulated by Alternative Splicing and Involved in Abiotic Stress Response
Source: Int J Mol Sci. 2020 Oct 26;21(21):7939. doi: 10.3390/ijms21217939 (PMC7663043; doi:10.3390/ijms21217939)
Supplement: Supplementary file 1 [file ijms-21-07939-s001.pdf]

Table. S1 The nucleotide sequences of *CML21* splice variants of *Vitis vinifera* and *Vitis amurensis*.

>VviCML21v1(VIT_219s0015g01200.1)
ATGGGAGGCGTGGTGGGAAAAGGCGATTCACCTAGAAAAGCCTGGGTACCAGAAACAAAGCTTGAGGCCA
AAATGGTTGAAGCTATGCAGCGGAGGGCATCTGAAGGAAGCACCATGAAATCGTTCAACAGTATAATCTTG
AAGTTCCCAAAAATTGATGAGAGTTTAAGGAACTGCAAAGCTATTTTTCAGCAATTTGATGAGGATTCAAAT
GGTGCAATAGATCATGAAGAACTGAAAAGATGTTTCCTTAAACTGGAGATTTCTTTTTCAGAGGAGGAAATC
AACGATCTTTTTGAGGCATGTGATATCAATGAGGATATGGGAATGAAGTTCAATGAGTTCATTGTACTTCTCT
GCCTTGTCTATCTTCTCAAGGAGGATCCTGCTGCCCTTCAGGCCGTATCCAAAAAATCACGGATGGGGATGC
CAAATCTGGAGGCAACCTTTGAAACACTGGTTGATGCATTTGTGTTCCTGGACAAGAATAAGGATGGTTATG
TGAGCAAGAGCGAGATGGTGCATGCAATAAATGAAACAACTGCAGGGGAGCGTTCTTCTGGAAGAATAGCC
ATGAAAAGATTCGAGGAGATGGATTGGGATAAAAATGGAATGGTGAACTTCAAGGAGTTTCTTTTTGCTTTC
ACCCGTTGGGTTGGGATTGACGATATTGAGGATGAAGAAGAAGAAGGTGAAGAGAAAGTTTGA
>VviCML21v2(VIT_219s0015g01200.2)
ATGGGAGGCGTGGTGGGAAAAGGCGATTCACCTAGAAAAGCCTGGGTACCAGAAACAAAGCTTGAGGCCA
AAATGGTTGAAGCTATGCAGCGGAGGGCATCTGAAGGAAGCACCATGAAATCGTTCAACAGTATAATCTTG
AAGTTCCCAAAAATTGATGAGAGTTTAAGGAACTGCAAAGCTATTTTTCAGCAATTTGATGAGGATTCAAAT
GGTGCAATAGATCATGAAGAACTGAAAAGATGTTTCCTTAAACTGGAGATTTCTTTTTCAGAGGAGGAAATC
AACGATCTTTTTGAGGCATGTGATATCAATGAGGATATGGGAATGAAGTTCAATGAGTTCATTGTACTTCTCT
GCCTTGTCTATCTTCTCAAGGAGGATCCTGCTGCCCTTCAGGCCAAATCACGGATGGGGATGCCAAATCTGG
AGGCAACCTTTGAAACACTGGTTGATGCATTTGTGTTCCTGGACAAGAATAAGGATGGTTATGTGAGCAAGA
GCGAGATGGTGCATGCAATAAATGAAACAACTGCAGGGGAGCGTTCTTCTGGAAGAATAGCCATGAAAAG
ATTCGAGGAGATGGATTGGGATAAAAATGGAATGGTGAACTTCAAGGAGTTTCTTTTTGCTTTCACCCGTTGG
GTTGGGATTGACGATATTGAGGATGAAGAAGAAGAAGGTGAAGAGAAAGTTTGA
>VviCML21v3(VIT_219s0015g01200.7)
ATGCTGTGTATCATCCTTCATGTTGTATTTGCTTCTTCCCTCTCTGACATGCCGGTGGTTGAAATTCATTGTTTT
TTCTTCATAGATGAGGATTCAAATGGTGCAATAGATCATGAAGAACTGAAAAGATGTTTCCTTAAACTGGAG
ATTTCTTTTTCAGAGGAGGAAATCAACGATCTTTTTGAGGCATGTGATATCAATGAGGATATGGGAATGAAG
TTCAATGAGTTCATTGTACTTCTCTGCCTTGTCTATCTTCTCAAGGAGGATCCTGCTGCCCTTCAGGCCGTATC
CAAAAAATCACGGATGGGGATGCCAAATCTGGAGGCAACCTTTGAAACACTGGTTGATGCATTTGTGTTCCT
GGACAAGAATAAGGATGGTTATGTGAGCAAGAGCGAGATGGTGCATGCAATAAATGAAACAACTGCAGGG
GAGCGTTCTTCTGGAAGAATAGCCATGAAAAGATTCGAGGAGATGGATTGGGATAAAAATGGAATGGTGAA
CTTCAAGGAGTTTCTTTTTGCTTTCACCCGTTGGGTTGGGATTGACGATATTGAGGATGAAGAAGAAGAAGGT
GAAGAGAAAGTTTGA
>VviCML21v4(VIT_219s0015g01200.8)
ATGCTGTGTATCATCCTTCATGTTGTATTTGCTTCTTCCCTCTCTGACATGCCGGTGGTTGAAATTCATTGTTTT
TTCTTCATAGATGAGGATTCAAATGGTGCAATAGATCATGAAGAACTGAAAAGATGTTTCCTTAAACTGGAG
ATTTCTTTTTCAGAGGAGGAAATCAACGATCTTTTTGAGGCATGTGATATCAATGAGGATATGGGAATGAAG
TTCAATGAGTTCATTGTACTTCTCTGCCTTGTCTATCTTCTCAAGGAGGATCCTGCTGCCCTTCAGGCCAAATC
ACGGATGGGGATGCCAAATCTGGAGGCAACCTTTGAAACACTGGTTGATGCATTTGTGTTCCTGGACAAGAA
TAAGGATGGTTATGTGAGCAAGAGCGAGATGGTGCATGCAATAAATGAAACAACTGCAGGGGAGCGTTCTT
CTGGAAGAATAGCCATGAAAAGATTCGAGGAGATGGATTGGGATAAAAATGGAATGGTGAACTTCAAGGA
GTTTCTTTTTGCTTTCACCCGTTGGGTTGGGATTGACGATATTGAGGATGAAGAAGAAGAAGGTGAAGAGAA
AGTTTGA
>VaCML21v2(MN540599)
ATGGGAGGCGTGGTGGGAAAAGGCGATTCACCTAGAAAAGCCTGGGTACCAGAAACCAAGCTTGAGGCCA
AAATGGTTGAAGCTATTCAGCGGAGGGCATCTGAAGGAAGCGCCATGAAATCGTCCAACAGTATAATCTTG
AAGTTCCCAAAAATTGATGAGAGTTTAAGGAACTGCAAAGCTATTTTTCAGCAATTTGATGAGGATTCAAAT
GGTGCAATAGATCATGAAGAACTGAAAAGATGTTTCCTTAAACTGGAGATTTCTTTTTCAGAGGAGGAAATC
AACGATCTTTTTGAGGCATGTGATATCAATGAGGATATGGGAATGAAGTTCAATGAGTTCATTGTACTTCTCT
GCCTTGTCTATCTTCTCAAGGAGGATCCTGCTGCCCTTCAGGCCAAATCACGGATGGGGATGCCAAATCTGG
AGGCAACCTTTGAAACACTGGTCGATGCATTTGTGTTCCTGGACAAGAATAAGGATGGTTATGTGAGCAAGA
GCGAGATGGTGCATGCAATAAATGAAACAACTGCAGGGGAGCGTTCTTCTGGAAGAATAGCCATGAAAAG
ATTCGAGGAGATGGATTGGGATAAAAATGGAATGGTGAACTTCAAGGAGTTTCTTTTTGCTTTCACCCGTTGG
GTTGGGATTGACGATATTGAGGATGAAGAAGAAGAAGGTGAAGAGAAAGTTTGA
>VaCML21v3(MW033207)
ATGCTGTGTATCATCCTTCATGTTGTATTTGCTTCTTCCCTCTCTGACATGCCAGAGGTTGAAATTCGTTGTTTT
TTCTTTGTAGATGAGGATTCAAATGGTGCAATAGATCATGAAGAACTGAAAAGATGTTTCCTTAAACTGGAG
ATTTCTTTTTCAGAGGAGGAAATCAACGATCTTTTTGAGGCATGTGATATCAATGAGGATATGGGAATGAAG

TTCAATGAGTTCATTGTACTTCTCTGCCTTGTCTATCTTCTCAAGGAGGATCCTGCTGCCCTTCAGGCCGTATC
CAAAAAATCACGGATGGGGATGCCAAATCTGGAGGCAACCTTTGAAACACTGGTCGATGCATTTGTGTTCCT
GGACAAGAATAAGGATGGTTATGTGAGCAAGAGCGAGATGGTGCATGCAATAAATGAAACAACTGCAGGG
GAGCGTTCTTCTGGAAGAATAGCCATGAAAAGATTCGAGGAGATGGATTGGGATAAAAATGGAATGGTGAA
CTTCAAGGAGTTTCTTTTTGCTTTCACCCGTTGGGTTGGGATTGACGATATTGAGGATGAAGAAGAAGAAGGT
GAAGAGAAAGTTTGA

>VaCML21v4(MW033208)

ATGCTGTGTATCATCCTTCATGTTGTATTTGCTTCTTCCCTCTCTGACATGCCAGAGGTTGAAATTCGTTGTTTT
TTCTTTGTAGATGAGGATTCAAATGGTGCAATAGATCATGAAGAACTGAAAAGATGTTTCCTTAAACTGGAG
ATTTCTTTTTCAGAGGAGGAAATCAACGATCTTTTTGAGGCATGTGATATCAATGAGGATATGGGAATGAAG
TTCAATGAGTTCATTGTACTTCTCTGCCTTGTCTATCTTCTCAAGGAGGATCCTGCTGCCCTTCAGGCCAAATC
ACGGATGGGGATGCCAAATCTGGAGGCAACCTTTGAAACACTGGTCGATGCATTTGTGTTCCTGGACAAGAA
TAAGGATGGTTATGTGAGCAAGAGCGAGATGGTGCATGCAATAAATGAAACAACTGCAGGGGAGCGTTCTT
CTGGAAGAATAGCCATGAAAAGATTCGAGGAGATGGATTGGGATAAAAATGGAATGGTGAACTTCAAGGA
GTTTCTTTTTGCTTTCACCCGTTGGGTTGGGATTGACGATATTGAGGATGAAGAAGAAGAAGGTGAAGAGAA
AGTTTGA
